# Characteristics and determinants of knowledge transfer policies at universities and public institutions in medical research—protocol for a systematic review of the qualitative research literature

**DOI:** 10.1186/s13643-015-0094-3

**Published:** 2015-08-19

**Authors:** Rosa Jahn, Olaf Müller, Kayvan Bozorgmehr

**Affiliations:** Institute for Public Health, Im Neuenheimer Feld 324, Heidelberg, Germany; Department of General Practice and Health Services Research, University Hospital Heidelberg, Voßstr.2, Heidelberg, 69115 Germany

**Keywords:** Medical research policy, Technology transfer, Knowledge transfer, University-industry collaboration

## Abstract

**Background:**

Universities, public institutions, and the transfer of knowledge to the private sector play a major role in the development of medical technologies. The decisions of universities and public institutions regarding the transfer of knowledge impact the accessibility of the final product, making it easier or more difficult for consumers to access these products. In the case of medical research, these products are pharmaceuticals, diagnostics, or medical procedures. The ethical dimension of access to these potentially lifesaving products is apparent and distinguishes the transfer of medical knowledge from the transfer of knowledge in other areas. While the general field of technology transfer from academic and public to private actors is attracting an increasing amount of scholarly attention, the specifications of knowledge transfer in the medical field are not as well explored. This review seeks to provide a systematic overview and analysis of the qualitative literature on the characteristics and determinants of knowledge transfer in medical research and development.

**Methods:**

The review systematically searches the literature for qualitative studies that focus on knowledge transfer characteristics and determinants at medical academic and public research institutions. It aims at identifying and analyzing the literature on the content and context of knowledge transfer policies, decision-making processes, and actors at academic and public institutions. The search strategy includes the databases PubMed, Web of Science, ProQuest, and DiVa. These databases will be searched based on pre-specified search terms. The studies selected for inclusion in the review will be critically assessed for their quality utilizing the Qualitative Research Checklist developed by the Clinical Appraisal Skills Programme. Data extraction and synthesis will be based on the meta-ethnographic approach.

**Discussion:**

This review seeks to further the understanding of the kinds of transfer pathways that exist in medical knowledge transfer as well as what factors lead to the adoption of one pathway over another. The aim is to provide evidence for political and academic actors designing policies for the translation of medical knowledge and public-private cooperation.

**Systematic review registration:**

PROSPERO CRD42015014241.

## Background

Universities and public institutions play an important role in the development of new technologies in medicine.^1^ A 2010 study examining all pharmaceuticals developed between 1998 to 2007 found that of those pharmaceuticals classified as scientifically novel, more than 30 % originated from universities and were later developed by pharmaceutical or biotech companies [1]. This illustrates the role the public sector and the interaction between private and public actors play in the development of new medicines and medical technologies. The nature of this interaction has a great impact not only on the kinds of medicines that are developed but also on how well they are accessible. For example, the first candidates for the HPV vaccines preventing cervical cancer, Gardasil, and Cervarix, were developed by public institutions and universities [2]. These were later licensed to Glaxo Smith Kline and Merck Sharpe and Dohme. As the licenses were exclusive, only these two companies had the right to develop and sell the resulting vaccines when they came to market in 2006 and 2009 [3]. The resulting lack of competition has led to high prices, making it difficult for poorer populations to afford vaccination. However, HPV disproportionately affects the world’s poor, with over 80 % of the cases occurring in developing countries [4].

As the case of the HPV vaccine illustrates, furthering the understanding of how universities and public institutions transfer their technologies is of great interest to public health professionals. However, while university technology transfer in general has attracted an increasing amount of scholarly attention, the transfer of knowledge in the development of new medical technologies specifically has rarely been addressed.

However, “anyone studying technology transfer understands just how complicated it can be” [5]. Defining the terms “technology” and “transfer” is the first challenge.

According to the “system’s view of technology” [6], technology can be described as a system of processes and products and the knowledge of their use and production. The process of innovation is an evolution of this system and its configuration. Basic principles serve as a guide for further development, and breakthroughs result from the culmination of prior, smaller changes to it. These then in turn form the basis for new developments in a continuous evolutionary process of innovation. This evolutionary process of innovation makes it difficult to demarcate individual technologies [6].

In addition to “technology”, the term “knowledge” has also been used, albeit without a clear distinction between the two terms. Sahal argues that whenever a technology is transferred, the knowledge of its use and its production process has to be transferred with it. Thus, “the knowledge base is inherent, not ancillary” [5]. For the purpose of this review, the broader term “knowledge” is used and defined to include “technology”. According to the “system’s view of technology”, any new knowledge, or minor modification of existing knowledge, is part of the innovation process, making it unnecessary to demarcate specific technologies [6]. Therefore, this review involves studies that address the exchange of any scientific knowledge generated by a researcher at a public institution or university.

The scope of the word “transfer” has been the subject of discussions as well. One definition commonly used in empirical studies is “transfer of physical devices, technological processes, or ‘know how’ from your organization to another” [7]. In medical research, two types of knowledge transfer can be discerned. The first consists of the utilization of basic medical research for the “development of new methods for diagnosis, therapy, and prevention and their first testing in humans”, while the second describes the “translation of results from clinical studies into everyday clinical practice and health decision-making”. [8–10] In this study, we address the first type of knowledge transfer. However, as the two processes are closely related, our conceptual findings might be relevant for the study of knowledge translation into practice as well.

A suggested model of “transfer” in the context of university research was developed by Bradley, Hayter, and Link [11]. Their “new model of university technology transfer” distinguishes two main types of technology transfer—formal transfer (through the technology transfer office) and informal transfer (informal exchange with colleagues, conferences, etc.). They argue that the scientist, as the originator of the knowledge, is central to the transfer process. He/she decides whether or not to declare an invention to the institution’s technology transfer office (TTO). If the researcher declares the invention, the TTO can decide whether to patent the discovery or not. If it chooses to patent, it markets the innovation to a third party or founds a new company to commercialize the invention, a so-called spin-off; if it does not patent the invention, the knowledge enters the public domain or is claimed by the scientist. However, new knowledge that is not declared by the scientist as an invention can be transferred informally, at conferences, through joint publications or informal meetings. According to Bradley, Hayter, and Link, these different transfer pathways can happen in various ways and often simultaneously [11]. However, this concept is limited in scope, as it only includes transfer processes that arise from a, usually patentable, discovery and ends with the adoption of a product. It does not take into account collaborative research, especially if it precedes discovery, and transfer processes that do not have the goal of commercializing a product. This gap is filled by the concept of academic engagement, which includes all “knowledge related collaboration by academic researchers with non-academic organisations” [12]. Academic engagement differs from the alternative view of technology transfer in so far as the focus is less on “transfer” and more on “collaboration” between institutions, often based on individual interaction. Its focus is broader, it does not stipulate an immediate financial objective but acknowledges that some collaborations aim at generating a more vague kind of utility. Combined, academic engagement and the alternative view of university technology transfer provide a comprehensive basis for analyzing knowledge exchange between public and private research entities. This study seeks to review the qualitative literature on the formal or informal transfer of medical knowledge from public and academic research institutions to private entities. We aim to improve the understanding of public-private knowledge transfer by addressing four key questions: What is the context in which knowledge transfer occurs? What are possible transfer pathways? What is the process by which a pathway is chosen? Who are the actors involved in the decision-making and what power do they have?

## Methods/design

This review will include studies that address any kind of formal or informal method to transfer knowledge created at public institutions or universities to the private sector; and the factors that determine which of the possible policies is adopted. However, the scope of this literature review is limited to qualitative studies. Qualitative research “is most revealing when the variables of greatest concern are unclear” [13]. The questions of what the possible knowledge transfer methods are and what determines which strategy scientists and university staff apply are therefore properly addressed through qualitative research.

### Search strategy

The search strategy aims at finding both published and unpublished studies. A three-step search strategy will be utilized. In the first step, keywords for the search have been developed based on the PICo approach. The PICo mnemonic has been developed for systematic reviews of qualitative literature, its components are population (P), phenomenon of interest (I), and context (Co). In comparison to quantitative reviews, it does not include an outcome, as “the expression of the phenomena of interest is the outcome.” [14] In the case of this review, scientists at universities and public research institutions represent the population, knowledge transfer, the phenomenon of interest, and medical research and development the context.

The databases to be searched are PubMed and Web of Science. The search for unpublished studies will include ProQuest and DiVA. Initial keywords included: research, development, medical, pharmaceutical, biomedical, university, academia, publicly funded, technology, innovation, results, discovery, knowledge, patent, transfer, translation, commercialization, transfer method, transfer pathway, transfer process, license, formal, informal. For full search terms, see Table 1. The keywords were used in a cursory search of PubMed, Web of Science, ProQuest, and DiVA. The first results of this limited search were evaluated for relevance and the search terms were refined accordingly and adapted to the respective database. In the second step, a full search of the databases was undertaken using the refined and prespecified search terms.

**Table 1:** Databases and search terms

Database:	Term:
PubMed	(medical OR medicine OR biomedical OR pharmaceutical OR biotech) AND (university OR academic OR universities OR facult* OR public institut*) AND (Technology OR innovation OR knowledge) AND (Transfer OR Translation OR commercialization OR License OR ((Joint OR collaborative OR contract) AND research)) AND (industry OR “private sector” OR company)
Web of Science	(medic* OR biomedical OR pharmaceutical* OR biotech) AND (universit* OR academic OR facult* OR publicly funded OR public institution) AND (Technology OR innovation* OR knowledge) AND (Transfer OR Translation Or commercialization OR License OR ((Joint OR collaborative OR contract) research)) AND (industry OR “private sector” OR company)
ProQuest	AB((medical OR biomedical OR pharmaceutical) AND (university OR academic* OR faculty* OR (public* NEAR/2 fund* NEAR/2 research) OR “public institution”) AND (Technology OR innovation) AND (Transfer OR Translation OR commercialization OR (University NEAR/5 Industry near/5 collabor*)) AND (industry OR “private sector” OR compan*))
DiVa	(medical OR biomedical OR pharmaceutical) AND (university OR academic OR faculty OR “publicly funded research” OR “public institution”) AND (Technology OR innovation) AND (Transfer ORTranslation OR commercialization OR collabor*) AND (industry OR “private sector”)
DART - Europe	(medical OR biomedical OR pharmaceutical) AND (university OR academic OR faculty OR “public institution”) AND (Technology OR innovation) AND (Transfer OR Translation OR commercialization OR collabor* OR industry OR “private sector”)

A pre-selection based on title and abstract will be identified by applying the inclusion and exclusion criteria. A random set of 10 % of the studies will be pre-selected in duplicate (RJ, KB) to test the criteria for inclusion and exclusion and establish a consensus on the selection procedure. The remainder of the studies will be pre-selected by one researcher (RJ), unclear cases will be discussed in the review team.

The research team then scans the full text articles, again applying the inclusion and exclusion criteria.

Studies published in German and English and between 1995 and 2014 will be considered for inclusion in this review. This is because the major international agreement regarding the protection of intellectual property, “Trade Related Aspects of Intellectual Property” (TRIPS), including medical inventions, entered into force on 1 January 1995 and led to a restructuring of technology transfer nationally and internationally [15].

The resulting studies will be critically appraised for their quality using the qualitative research checklist developed by the Critical Appraisal Skills Programme (CASP) [16], and data will be extracted. The data will be analyzed using the meta-ethnographic approach. To ensure relevance to policy makers, the synthesis will be framed in accordance with the health policy triangle, a policy analysis framework developed by Gill Walt and Lucy Gilson (1994).

### Inclusion criteria

The systematic review will include primary research studies that focus on qualitative data, cover technology transfer policies at universities or public institutions and address at least one of the following: a) content – possible technology transfer policies (spin-off, types of licenses, informal transfer, etc.), b) context – individual or institutional determinants of the adoption of technology transfer policies, c) process – procedural characteristics of decision-making regarding technology transfer, and d) actors – information about parties involved in the decision-making regarding technology transfer. The studies must also cover medical research as a single study subject or within a range of study subjects, (medical meaning pharmaceuticals, diagnostics, medical devices and procedures), have an empirical or systematic approach and be peer reviewed or dissertations.

### Exclusion criteria

The systematic review will exclude works that are commentaries, theoretical texts, books, and meeting reports that do not specifically address medical research and development or focus on an industry perspective or on effectiveness and performance of technology transfer.

### Critical quality appraisal

Qualitative papers selected for retrieval will be assessed by two independent reviewers for methodological rigour using the qualitative research checklist developed by CASP in 2012 [16]. It evaluates theoretical approach, study design, data collection, data analysis, and ethics on the basis of ten questions. Any disagreements in grading that arise between the reviewers will be resolved through discussion. The score (1–10) of each included study will be indicated in the final report, and the possible influence of quality issues on the overall synthesis will be discussed.

### Data extraction and synthesis

Data extraction and synthesis will follow the meta-ethnography approach first developed by Noblit and Hare. It describes an approach whereby major themes, “metaphors” are identified and then compared across studies. They describe three major strategies, of which we will follow the “lines-of-argument synthesis” (LOA). This approach is appropriate in cases where “many studies suggest a lines-of-argument or inference about some larger issue or phenomenon” [17]. It allows for the synthesis of studies that are diverse and offer an illustration of different aspects of a larger “whole”. This is likely to be the case in this study because the included studies are going to cover various aspects of knowledge transfer. Synthesis “involves building a general interpretation grounded in the findings of the separate studies” [18]. The findings, or key metaphors, will be identified using open coding in MAXQDA to allow for systematic analysis of a larger number of studies and facilitate oversight. After extracting the data of the first two studies, the review team will evaluate and if necessary revise the extraction strategy. The coded metaphors will be grouped according to their content so related metaphors can be analyzed and translated into each other across studies. These translations and their relationships will be reported in the synthesis. Data extraction and synthesis will be done in duplicate. The synthesis of the systematic review should not just be well-grounded in theory, but it should also be relevant and appropriate for policy makers. Embrett and Randall have examined the health and health equity policy literature. They have stated that issues raised by scholars in this field rarely make it to the policy agenda, identifying the common misuse and nonuse of political analysis theory as one of the reasons [19]. Therefore, this study uses the health policy triangle as a policy analysis tool to ensure the synthesized evidence will be relevant and useful to policy makers.

The health policy triangle is a policy analysis tool developed by Gill Walt and Lucy Gilson. It states that the adoption of policies depends on four aspects: context, content, process and actors [20]. In the context of this review, these aspects are:

- Context: What is the context in which knowledge transfer occurs?
- Content: What are possible technology transfer policies?
- Process: How is the transfer policy negotiated within the institution and with external partners?
- Actors: Who is involved in the decision-making at the university / public institution?

The health policy triangle will be used to frame the synthesis in a meaningful way. Its categories are broad enough to accommodate all findings and categories that might emerge from the data and can therefore be applied in conjunction with the meta-ethnographic approach.

## Discussion

This review seeks to further the understanding of the kinds of transfer pathways that exist in medical knowledge transfer as well as what factors lead to the adoption of one pathway over another. The aim is to provide evidence for political and academic actors designing policies for the translation of medical knowledge and public-private cooperation. Understanding the importance of technology transfer and its effect on access to medicines and equality in health, in conjunction with improved knowledge of how this transfer comes about and why might aid individual, institutional and political actors in shaping a research environment that is conducive to global health.

## Abbreviations

HPV: Human papilloma virus
CASP: Clinical Appraisal Skills Programme

## Footnotes

1 For the purpose of this study, the term “public research institution” includes all medical research entities primarily funded from public sources.
